# Participation of the nucleus accumbens dopaminergic system in the antidepressant-like actions of a diet rich in omega-3 polyunsaturated fatty acids

**DOI:** 10.1371/journal.pone.0230647

**Published:** 2020-03-25

**Authors:** Eri Takeuchi, Daisuke Yamada, Satoshi Suzuki, Akiyoshi Saitoh, Masayuki Itoh, Takashi Hayashi, Mitsuhiko Yamada, Keiji Wada, Masayuki Sekiguchi

**Affiliations:** 1 Department of Degenerative Neurological Diseases, National Institute of Neuroscience, National Center of Neurology and Psychiatry, Tokyo, Japan; 2 Department of Neuropsychopharmacology, National Institute of Mental Health, National Center of Neurology and Psychiatry, Tokyo, Japan; 3 Department of Biochemistry and Cellular Biology, National Institute of Neuroscience, National Center of Neurology and Psychiatry, Tokyo, Japan; 4 AMED-CREST, Japan Agency for Medical Research and Development, Tokyo, Japan; University of Kentucky, UNITED STATES

## Abstract

The beneficial effects of omega (ω)**-**3 polyunsaturated fatty acid (PUFA) supplementation on major depressive disorder have been actively studied, but the underlying mechanism remains unknown. The present study examined the involvement of the nucleus accumbens (NAc) dopaminergic systems in behavioral changes in mice fed a diet high in ω**-**3 PUFAs. Mice fed a diet containing about double the amount of ω**-**3 PUFAs (krill oil (KO) diet) exerted shorter immobility times in the forced swim test (FST) than mice fed a control diet, containing only α-linolenic acid (ALA) as ω**-**3 PUFAs. The shorter immobility times were observed in both male and female mice. A dopamine metabolite, 3,4-dihydroxyphenylacetic acid, increased in the NAc in male mice fed the KO diet when compared with those fed the control diet. In addition, dopamine, 3-methoxytyramine, and homovanillic acid increased in the NAc in female mice fed the KO diet. Notably, the effects of the KO diet on the immobility time in the FST were abolished by microinjection of sulpiride, an antagonist of D_2_**-**like receptors, into the NAc. A similar microinjection of an antagonist selective for D_1_-like receptors, SKF83566, also abolished the reduction in immobility in the FST. Moreover, we found that tyrosine hydroxylase-positive cells increased in the ventral tegmental area (VTA) in mice fed the KO diet. These results suggest that modulation of the VTA**-**NAc dopaminergic pathway is one of the mechanisms by which a KO diet rich in ω**-**3 PUFAs reduces the immobility behavior in the mouse FST.

## Introduction

In the mammalian brain, long-chain polyunsaturated fatty acids (PUFAs) are major plasma membrane components affecting membrane fluidity [1, 2]. The fatty acid composition of the brain is extensively affected by the dietary lipid composition [3]. Two major classes of PUFAs—omega (ω)**-**3 and ω**-**6, which are named according to the double bond position—are utilized in mammals. A large body of evidence has shown the beneficial effects of ω**-**3 PUFAs on human health [4–6]. Regarding mental health, an overall beneficial effect of ω-3 PUFA supplementation on major depressive disorders has been suggested by various clinical studies investigating the role of ω-3 PUFAs in the treatment of depression [7–10], and a recent meta-analysis involving 1 233 participants from 13 studies presented similar results [11]. However, publication bias has been pointed out in one study [12], and the mechanisms underlying the effect of ω-3 PUFAs on depression are still unclear.

In rodents, diets rich in ω-3 PUFAs attenuate immobility in the forced swim test (FST) [13–15], a frequently used screening test for antidepressant drugs [16]. Although the mechanism of the effect of ω-3 PUFAs on immobility in the FST is unclear, the retinoid X receptor (RXR) gamma and serotonin 5-hydroxytryptamine 1A (5-HT1A) receptors have been suggested to contribute to the effects of docosahexaenoic acid (DHA) and fish oil on immobility time in the FST, respectively [17, 18]. In addition, a change in the nucleus accumbens (NAc) dopaminergic system has been observed under α-linolenic acid (ALA) and other ω-3 PUFA dietary deficiency conditions throughout two generations of rodents—the tested mice and their mothers [19–22]. Dopaminergic systems have been shown to be related to ω-3 PUFA deficiencies and major depression in a clinical study [23]. Mammals can synthesize other ω-3 PUFAs, such as eicosapentaenoic acid (EPA) and DHA, from ALA alone, although inefficiently. However, mammals cannot self-autonomously synthesize ALA; therefore, ALA must be ingested from food, and ALA-deficient diets lead to a severe ω-3 PUFA deficiency, resulting in malnutrition. In the FST, the immobility time is significantly longer in ALA-deficient mice than in non-deficient ones [24]. Under normal nutrient conditions, however, ALA is usually sufficiently supplied as it is abundantly available in popular edible oils, such as soybean and canola oil. Therefore, elucidating the mechanism underlying the antidepressant-like actions of ω-3 PUFA supplementation in the ALA-sufficient condition is required to understand the effects of ω-3 PUFAs in the treatment of depression.

Krill (*Euphausia superba*) is a small crustacean. Krill oil (KO) extracted from Antarctic krill contains a high concentration of ω-3 PUFAs, particularly EPA and DHA. Fish oil contains EPA and DHA in the triglyceride form, whereas in KO they are mainly in the phospholipid form [25, 26]. The antidepressant-like effects of KO intake have previously been reported in a study in rats, wherein the immobility time was significantly reduced after 7 weeks of supplementation with KO [27]. However, the mechanism(s) of these antidepressant-like effects are still unclear.

In the present study, we compared the immobility time in the FST between young adult mice (< 13 weeks old) fed low or high ω-3 PUFA-containing diets. The low ω-3 PUFA-containing diet (control diet) was prepared using only soybean oil (7%, Table 1), which contained only 7.6% ALA as ω-3 PUFAs (of the total fatty acids, Table 2). The high ω-3 PUFA-containing diet (KO diet) was prepared using soybean oil (2%) and KO (5%), which contained 16.4% ω-3 PUFAs (ALA, 3.3%; 18:4n-3, 2.1%; EPA, 9.2%; DHA, 4.5%). Considering that the prevalence of major depression is higher in females than in males, this investigation was performed on both male and female mice in some experiments [28]. The immobility time was markedly reduced in mice fed the KO diet compared to mice fed the control diet. In addition, we compared the levels of dopamine, serotonin, norepinephrine, and corresponding metabolites in the medial prefrontal cortex (mPFC), NAc, hippocampus (HPC), and amygdala (AMY) of mice that were fed different diets. Moreover, we examined the effects of a dopamine antagonist injected into the NAc on the KO diet-induced reduction in FST-immobility time. Finally, we compared immunohistochemical *staining in the control and KO diet mice* for tyrosine hydroxylase (TH) in the ventral tegmental area (VTA), which is the site for NAc dopamine cell bodies.

**Table 1:** Nutritional information of the diets used.

	Control diet	KO diet
Milk caseins	20.000	20.000
L-Cysteine	0.300	0.300
Corn starch	39.247	39.247
Pregelatinized starch	13.200	13.200
Sucrose	10.000	10.000
Cellulose powder	5.000	5.000
AIN-93G mineral mixture	3.500	3.500
AIN-93 vitamin mixture	1.000	1.000
Choline bitartrate	0.250	0.250
ter-Butylhydroquinone	0.001	0.001
Soybean oil	7.000	2.000
Krill oil	0.000	5.000

Value = g/100 g

**Table 2:** Composition of fatty acids (FAs) in the diet.

Numerical symbol	Control (7% Soybean oil)	KO (2% Soybean oil + 5% Krill oil)
total saturated FA	16.1	31.1
total monounsaturated FA	22.9	25.4
18:3n-3 (ɑ-linolenic acid, ALA)	7.6	3.3
18:4n-3	-	2.1
20:5n-3 (eicosapentaenoic acid, EPA)	-	9.2
21:5n-3	-	0.3
22:5n-3	-	0.3
22:6n-3 (docosahexaenoic acid, DHA)	-	4.5
18:2n-6 (linoleic acid, LA)	52.5	20.2
20:4n-6 (arachidonic acid, AA)	-	0.2
unknown	0.9	3.4

Value: % of the total fatty acids

## Materials and methods

### Animals

Male and female C57BL/6JJcl mice, aged 4–5 weeks, were purchased from CLEA Japan, Inc. (Tokyo, Japan) and fed a solid standard mouse diet (CE-2; CLEA Japan) for 1 week. Subsequently, the mice received test diets for 6–8 weeks before performing the behavioral or biochemical experiments. The mice were housed three to five per cage under controlled temperature (25 ± 1°C) and lighting (12 h light/dark cycle) conditions, with food and water *ad libitum*. The animal procedures were *conducted* in strict accordance with the guidelines of the National Institute of Neuroscience, National Center of Neurology and Psychiatry (Tokyo, Japan) and were approved by the Institutional Animal Investigation Committee (approval number: 2017012). All efforts were made to minimize both suffering and the number of animals used.

### Diets

The control and KO diets were prepared in our laboratory [29], and the basic compositions of these two diets were the same, as previously reported in the introduction. The control diet was similar to the standard diet AIN-93, and soybean oil was used to provide the fat component. The KO diet was prepared by partially replacing soybean oil with KO (rich in ω-3 PUFA), prepared from Antarctic krill. The oil was mixed with the other chow components before solidification [29]. Therefore, the total fat contents did not differ between the diets used. The nutritional information and composition of the fatty acids in the diets are presented in Tables 1 and 2.

### Forced swim test (FST)

After the defined feeding period, the male and female mice were subjected to the FST. Mice were put in a glass beaker (12 cm in diameter, 25 cm high) filled two-thirds with water at 25 ± 1°C for 6 min. The duration of immobility was videotaped and scored manually, and this parameter was analyzed during the last 4 min of the FST by two trained experimenters. To further confirm the effect of diets, the KO diets were changed to the control diet after 7 weeks of feeding, and the control diet was supplied for 4 weeks. In this trial, we tested the first FST at 6 weeks of feeding. The second FST was carried out after additional 4 weeks of feeding with the control diet. Regarding the pharmacological experiments, male mice were administered sulpiride (SUL; 25 ng/mouse) (a dopamine D_2_-like receptor antagonist), SKF83566 (SKF; 0.264 ng/mouse) (a D_1_-like receptor antagonist), or a vehicle (VEH) into the NAc 15 min before the FST. New animal groups were used for each trial unless otherwise stated.

### Tail suspension test (TST)

We performed a further test to assess the antidepressant-like behavior through a TST according to published methods [30] using only the male mice. A clear acrylic tubing cut 4 cm in length (1.6 cm outside diameter, 1.2 cm inside diameter, 3.8 g) was placed around the tails of mice to prevent them from climbing their tails. The tails of the mice were attached to a bar with tape and the mice were suspended for 6 min. The activity was recorded using a video camera, and the duration of immobility scored manually. The parameter was analyzed for 6 min. If the mouse was able to climb its own tail, the data was excluded.

### Barnes maze test

After the 6-week feeding period, male mice were tested in the Barnes maze test as previously described with slight modifications [31]. A Barnes maze has a circular platform (1 m diameter) illuminated by bright lighting and 12 equally spaced holes. A black plastic box (escape box, 17 × 13 × 7 cm) was placed under the target hole and the position of target was changed for each animal. Visual cues were set around the maze, and mice sought the position of the target hole using the visual cues to avoid the bright light. The behavior of the mice was recorded by a video camera above the platform. Three trials per day were conducted on four successive days. On day five, a probe test was conducted without the target box to check whether this spatial task was performed based on navigation by the distal cues in the room. The latency to reach the target hole and the time spent around each hole were recorded by the video tracking Time BCM software (O’Hara & Co., Ltd., Tokyo, Japan).

### Three-chamber social interaction test

The test for sociability and social memory (preference for social novelty) in the male mice were conducted using a three-chambered social interaction test as previously described [32, 33]. Three interconnected chambers (610 × 400 × 220 mm) were placed in a soundproof box (O’Hara & Co., Ltd., Tokyo, Japan), and each chamber divided by clear panels with a hole to allow mice free access to each chamber. Wired cages were then placed in the corner of each side chamber. During habituation, empty wired cages were placed in both the right and left chambers, and a subject mouse was placed in the middle chamber. The subject mouse behavior was then monitored for 10 min. During the sociability test, an unfamiliar mouse (stranger 1) was placed in one wired cage, while the other cage was left empty. The behavior of the subject mouse was monitored for 10 min. The amount of time that the subject mouse spent in each chamber was measured. Moreover, the amount of time that the subject mouse stayed around each wired cage was measured and then the ratio of the time that mouse stayed in the stranger 1 cage/empty cage (referred to as preference index of sociability here) was calculated. During the social novelty preference test, the stranger 1 and a new, unfamiliar mouse (stranger 2) were placed in the wired cage, and the behavior of the subject mouse was again monitored for 10 min. The amount of time spent in each chamber was measured to clarify the preference of stranger 1 compared with that of stranger 2. Furthermore, the amount of time that the subject mouse stayed in each wired cage was measured, and the ratio of the time it stayed in the stranger 2 cage/stranger 1 cage (referred as the preference index of social novelty preference here) was calculated. Analyses were performed automatically using Image CSI software (O’Hara & CO., Ltd., Tokyo, Japan).

### Quantification of monoamines and their metabolites

High-performance liquid chromatography (HPLC) was used to quantify monoamines and associated metabolites, as previously described [34], in four brain regions (AMY, HPC, mPFC, and NAc) in the remaining male and female mice that were not used for the behavioral testing. The brain tissues were homogenized in 300 μL of 0.2 M perchloric acid containing 100 μM EDTA-2 Na, and 10 ng of isoproterenol was added as an internal standard. To remove the proteins completely, the homogenates were placed in cold water for 30 min and then centrifuged at 13 000 g for 15 min at 0°C. The upper layer was maintained at pH 3.0 using 1 M sodium acetate. A total of 10 μL samples were analyzed by HPLC via electrochemical detection. The electrochemical detector (HTEC-700; Eicom Co., Kyoto, Japan) was equipped with a graphite electrode (WE-3G; Eicom Co., Kyoto, Japan) that was used at a voltage setting of 750 mV with an Ag/AgCl reference electrode. The mobile phase consisted of a 0.1 M sodium acetate/0.1 M citric acid buffer (pH 3.5) containing 17% methanol, 190 mg/L sodium 1-octanesulfonate, and 5 mg/L EDTA-2 Na. Monoamines were separated on a C-18 column (150 mm × 3 mm reversed-phase, EICOMPAK SC- 5ODS, Eicom Co.). The mobile phase flow rate was maintained at 0.5 mL/min with a column temperature of 25°C.

### Stereotaxic surgery and intra-NAc infusion

Each male mouse (9–10 weeks old) was anesthetized with ketamine (100 mg/kg, i.p.) and xylazine (30 mg/kg, i.p.) and affixed to the brain stereotaxic apparatus (NARISHIGE, Tokyo, Japan). Guide cannulas (Plastics One, Roanoke, VA) were bilaterally implanted into the brain so that their tips were positioned near the NAs (AP: + 1.2 mm, ML: ± 0.6 mm, DV: -3.8 mm from Bregma) [35] and were fixed to the skull with dental cement. A dummy cannula was inserted into each guide cannula to prevent clogging. A microinjection (0.2 μL/one side) of SUL (12.5 ng/0.2 μL), SKF (0.132 ng/0.2 μL) or vehicle (saline for both drugs) was performed on day 7 after surgery. For the microinjections, the dummy cannula was removed from the guide cannula, and a 33-gauge injection cannula, extending 1 mm from the tip of the guide cannula, was inserted without anesthesia. The injection cannula was connected via Teflon tubing to a microsyringe (Hamilton Company, Reno, NV) driven by a microinfusion pump (KD Scientific Inc., MA, USA). The injection rate was 0.2 μL/min. The injection cannula was left in position for an additional 2 mins before withdrawal. The FST was performed 15 mins after injection. After the behavioral tests, injection sites were examined. If the injection site was out of NAc, the data of the individual was excluded.

### Drugs

Sulpiride was purchased from Wako Pure Chemical Industries, Ltd. (Osaka, Japan), and SKF83566 hydrobromide was purchased from Abcam (Tokyo, Japan). The other chemicals used were purchased from Sigma-Aldrich (Tokyo, Japan).

### Immunohistochemistry

After 6 weeks of consuming the KO or control diet, the male mice were transcardially perfused with phosphate buffer saline (PBS) and 4% paraformaldehyde under sevoflurane anesthesia. The brains were removed from the skull and postfixed overnight in 4% paraformaldehyde. The coronal sections (50 μm thickness) were prepared with a vibratome (Dosaka EM Co., Ltd., Kyoto, Japan). The slices were blocked in PBS containing 5% normal goat serum (Vector Laboratories, Burlingame, CA) and 0.3% Triton X-100 for 1 h at room temperature and were then incubated with an anti-TH antibody (1:1200 dilution; Cell Signaling Technology; #13106) overnight at 4°C. After washing with PBS, the slices were incubated with Alexa Fluor 488 goat anti-rabbit IgG (1:500 dilution; life technologies) at room temperature for 1 h. The slices were washed with PBS, after which they were mounted on microscope slides. The staining was photographed by a digital microscope (KYENCE CORPORATION, Osaka, Japan). The number of TH-positive cells in the same circular shape area of the VTA at the level of the bregma at -2.91 mm, -3.07 mm, and -3.15 mm [35] was counted manually. The area was set beside the fasciculus retroflexus. TH-positive cells in the bilateral VTA of two consecutive slices from one animal were counted.

### Statistical analysis

All data are presented as mean and standard error of the mean (SEM). All data were analyzed using the GraphPad Prism version 7 (GraphPad Software Inc., CA, USA). A two-tailed unpaired *t*-test or Mann-Whitney *U*-test was used for statistical comparisons between the two groups. Student’s *t*-test was used for homoscedastic data, and Welch’s *t*-test was used for heteroscedastic data. The data were analyzed by a one-way analysis of variance (ANOVA), two-way ANOVA and two-way repeated measures ANOVA among three or more groups. If the ANOVA results were significant, *post hoc* Bonferroni’s multiple comparisons were performed. p < 0.05 was considered statistically significant. To measure the effect size, the *r* value was calculated using the following formula.

When the unpaired *t*-test was used, the *r* value was given by:

$$r=\sqrt{\frac{t^{2}}{t^{2}+df}}$$

When Mann-Whitney *U*-test was used, the *r* value was given by:

$$r=\frac{Z}{\sqrt{N}}$$

z value, E (*U*) and V (*U*) are given by:

$$z=\frac{\left|U-E(U)\right|}{\sqrt{V(U)}}$$

$$E\;(U)=\frac{n_{1}\times n_{2}}{2}$$

$$V\;(U)=\frac{n_{1}{\times n}_{2}\;(n_{1}+n_{2}+1)}{12}$$

n represented the number of samples.

## Results

### KO diet affects depression-like behavior

**Fig 1:**
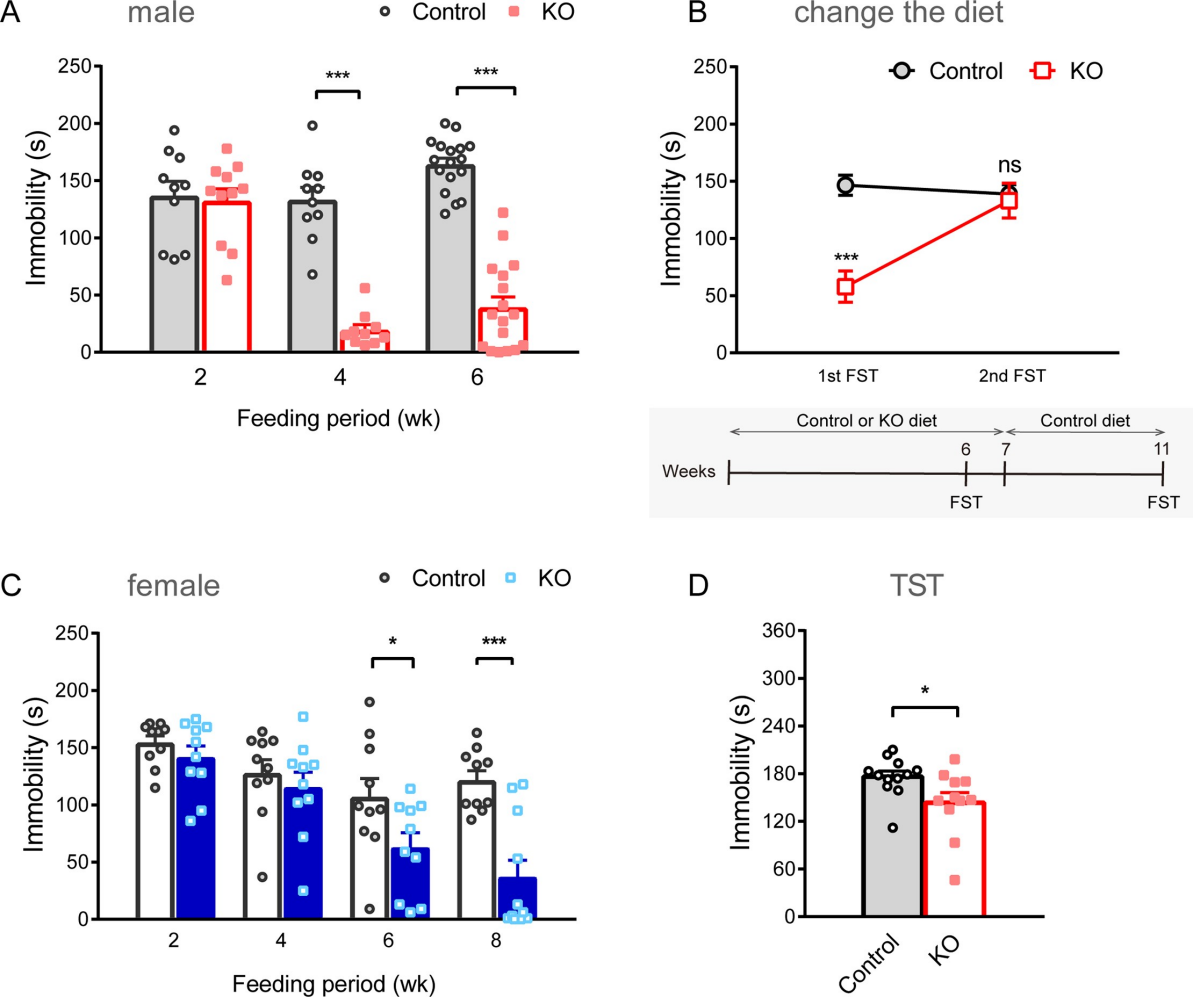
Antidepressant-like effects of KO diet in mice. (A) Antidepressant-like effects of a KO diet on FST in the male mice. Male mice fed a KO diet exhibited reduced FST-immobility times when compared with those fed the control diet. A comparison of the antidepressant-like effects of the KO diet in male mice at different feeding periods showed that consuming a KO diet for 4 weeks resulted in antidepressant-like effects. (B) The reversible effect of the KO diet. The reduction in the immobility time was eliminated after the KO diet was changed to the control diet. Two-way repeated measures ANOVA was used for comparison. (C) Antidepressant-like effect of the KO diet in female mice. Female mice fed the KO diet also exhibited reduced immobility times when compared with female mice fed a control diet. Ingestion of a KO diet for 6 weeks showed antidepressant-like effects in female mice. (D) Antidepressant-like effects of the KO diet on TST. A KO diet reduced the TST-immobility time in male mice, compared to a control diet. One individual of the KO diet group was excluded from TST data as it climbed its tail. All data are presented as mean and SEM. *: p < 0.05, ***: p < 0.001 Different animals were used for each behavioral trial.

We first examined whether supplementation of ω-3 PUFAs affected FST-immobility in male mice (Fig 1A). Male mice fed a KO diet for 6 weeks showed less immobility time than male mice fed a control diet (*t* [32] = 11.56, p < 0.001, *r* = 0.90, *n* = 17 for each group), as shown in a previous study using 7 weeks of KO supplementation in rats [27]. The effects of the KO diet on immobility were not evident in the 2-week feeding group (*t* [19] = 0.2648, p = 0.794, *r* = 0.06; control, n = 10; KO, n = 11), but they were clearly observed in the 4-week feeding group (*U* [10, 10] = 0, p < 0.001, *r* = 0.85), suggesting time-dependent effects of ω-3 PUFA supplementation on immobility. The reduction in time of FST-immobility disappeared when the diet was changed to the control diet after a 7-week ingestion period of the KO diet (diet: *F* [1, 19] = 12.56, p < 0.01; treatment: *F* [1, 19] = 9.519, p < 0.01; diet × treatment: *F* [1, 19] = 14.36, p < 0.01; two-way ANOVA repeated measured; before changing diet: control vs. KO, p < 0.001, after changing the diet: control vs. KO, p > 0.999, *post hoc* Bonferroni comparisons; Fig 1B), indicating that the effect of the KO diet on immobility was reversible.

Considering that the prevalence of major depression is higher in females than in males, we conducted similar experiments in female mice separately (Fig 1C). Female mice fed a KO diet for 6 weeks showed less immobility time (*t* [18] = 2.117, p = 0.048, *r* = 0.45 *n* = 10 for each group) and the 8-week feeding group also exhibited a reduction in immobility time (*t* [19] = 4.804, p < 0.001, *r* = 0.74; control, n = 10; KO, n = 11) compared to female mice fed a control diet, whereas the effects were not observed in the 2-week feeding group (*t* [18] = 1.083, p = 0.293, *r* = 0.25, *n* = 10 for each group) and 4 week feeding group (*U* [10, 10] = 38, p = 0.382, *r* = 0.20). To compare the effect size, male mice in the 4-week and 6-week feeding group showed a large effect, while the female 6-week and 8-week feeding groups showed a medium and large effect, respectively. These results suggest that ω-3 PUFA supplementation reduces immobility time in both male and female mice in a time-dependent feeding manner. We confirmed the antidepressant-like action of the KO diet using another behavioral test in male mice. The KO diet also induced the reduction of immobility time in the TST (*U* [12, 11] = 26.5, p < 0.05, *r* = 0.51; Fig 1D).

### KO diet specifically altered brain levels of dopamine and related metabolites

Next, we quantified the levels of monoamines and associated metabolites in four brain regions (AMY, HPC, mPFC, and NAc) considered to be involved in the pathophysiology of depression (Tables 3 and 4, S1 Table and S2 Table). A tendency of increased dopamine (*t* [29] = 2.106, p = 0.088) and related metabolite levels, such as homovanillic acid (HVA) (*t* [29] = 1.792, p = 0.084), was observed in the NAc of brain samples from male mice fed the KO diet (*n* = 16 for all brain regions tested) compared with male mice fed a control diet (*n* = 15 for all brain regions tested) (Table 3). Another dopamine metabolite, 3,4-dihydroxyphenylacetic acid (DOPAC), significantly increased in the NAc of male mice fed the KO diet (*t* [29] = 2.106, p < 0.05). No significant changes in other monoamines (serotonin and norepinephrine) and their metabolites were observed in all four brain regions tested. Dopamine levels significantly increased in the NAc of female mice fed a KO diet (*U* [16, 16] = 72, p *<* 0.05, Table 3) compared to those fed a control diet (each group, n = 16). Moreover, 3-methoxytyramine (3-MT) and HVA significantly increased in the NAc of female mice fed a KO diet compared to those fed a control diet (3-MT: *U* [16, 16] = 63, p < 0.05; HVA: *U* [16, 16] = 64, p < 0.05; Table 3). Other monoamines and their metabolites did not significantly differ between these two groups of female mice. No significant changes in monoamines were observed in the mPFC, HPC, and AMY in both male and female mice (S1 Table). The turnover ratio of 3-MT/DA was significantly increased in male mice of the KO diet feeding group (Table 4). In female mice, the turnover ratios of DOPAC/DA and 5-HIAA/5-HT were significantly increased (Table 4). Furthermore, no significant changes in turnover ratio were observed in the mPFC, HPC, and AMY in both male and female mice (S2 Table).

**Table 3:** Levels of monoamine in the NAc.

**Male**				
	Control	KO	p	
DA	1.003 ± 0.213	1.535 ± 0.213	**0.088**	
DOPAC	0.160 ± 0.024	0.248 ± 0.033	**0.044**	*
3-MT	0.046 ± 0.009	0.067 ± 0.010	0.127	
HVA	0.272 ± 0.025	0.335 ± 0.025	**0.084**	
5-HT	0.473 ± 0.027	0.527 ± 0.029	0.191	
5-HIAA	0.191 ± 0.015	0.217 ± 0.027	0.711	
NE	0.255 ± 0.013	0.291 ± 0.022	0.180	
MHPG	0.073 ± 0.012	0.066 ± 0.004	0.232	
**Female**				
	Control	KO	p	
DA	2.188 ± 0.361	3.354 ± 0.353	**0.035**	*
DOPAC	0.452 ± 0.051	0.551 ± 0.053	0.138	
3-MT	0.145 ± 0.022	0.213 ± 0.023	**0.014**	*
HVA	0.504 ± 0.033	0.638 ± 0.048	**0.015**	*
5-HT	0.702 ± 0.040	0.774 ± 0.056	0.254	
5-HIAA	0.412 ± 0.020	0.394 ± 0.026	0.239	
NE	0.317 ± 0.023	0.275 ± 0.019	0.138	
MHPG	0.094 ± 0.006	0.092 ± 0.007	0.841	

Value: ng/mg tissue

Mean ± SEM

**Table 4:** Monoamine turnover rates in the NAc.

**Male**				
	Control	KO	p	
DOPAC / DA	0.281 ± 0.057	0.175 ± 0.018	0.175	
3-MT / DA	0.061 ± 0.008	0.042 ± 0.002	**0.037**	*
HVA / DA	0.726 ± 0.229	0.260 ± 0.023	0.163	
5-HIAA / 5-HT	0.403 ± 0.024	0.396 ± 0.029	0.711	
MHPG / NE	0.313 ± 0.068	0.236 ± 0.012	0.654	
**Female**				
	Control	KO	p	
DOPAC / DA	0.254 ± 0.023	0.169 ± 0.007	**0.003**	*
3-MT / DA	0.074 ± 0.006	0.064 ± 0.003	0.361	
HVA / DA	0.337 ± 0.056	0.201 ± 0.011	0.094	
5-HIAA / 5-HT	0.599 ± 0.026	0.513 ± 0.014	**0.021**	*
MHPG / NE	0.307 ± 0.018	0.341 ± 0.016	0.166	

Mean ± SEM

### NAc dopamine D_1_-like and D_2_-like receptors are involved in the modulation of depression-like behavior via KO diets

**Fig 2:**
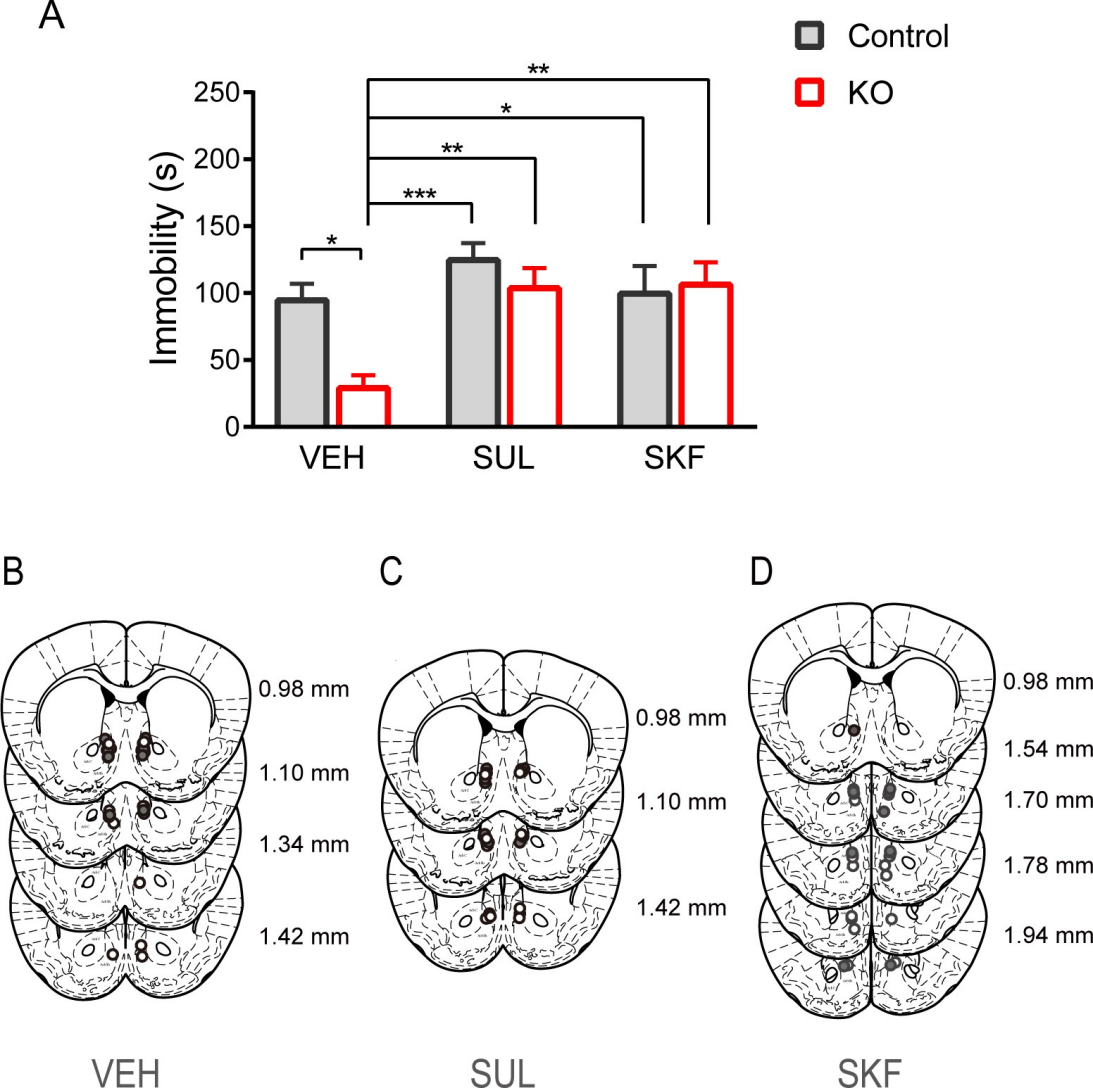
Participation of D_1_- and D_2_-like receptors in antidepressant-like effects of the KO diet. (A) Effects of the dopamine D_2_-like and D_1_-like receptor antagonist on FST-immobility time in the male mice. All data are presented as mean and SEM. *: p < 0.05, **: p < 0.01, ***: p < 0.001 (B) Schematic diagram showing the injection site of the vehicle. Each circle indicates a different injection site (white: control + VEH; grey: KO + VEH). (C) Schematic diagram showing the injection site of sulpiride. Each circle indicates a different injection site (white: control + SUL; grey: KO + SUL). (D) Schematic diagram showing the injection site of SKF83566. Each circle indicates a different injection site (white: control + SKF; grey: KO + SKF).

Since dopamine metabolism was altered in the NAc, we next determined whether dopamine receptors are involved in the antidepressant-like effect of the KO diet using pharmacological experiments in the male mice. Each mouse was injected with the dopamine D_2_-like receptor antagonist SUL (25 ng/mouse), the D_1_-like receptor antagonist SKF (0.264 ng/mouse) or VEH into the NAc 15 min before the FST. Two-way ANOVA revealed interaction between diet and treatment (diet: *F* [1, 47] = 5.153, p < 0.05; treatment: *F* [2, 47] = 8.018, p < 0.01; diet × treatment: *F* [2, 47] = 3.454, p < 0.05; two-way ANOVA). Mice in the KO diet plus VEH group (KO + VEH, *n* = 11) had significantly less immobility time than mice in the control diet plus VEH group (control + VEH, *n* = 10; p < 0.05, *post hoc* Bonferroni comparisons; Fig 2A). However, the reduction in immobility time in mice fed the KO diet was abolished by intra-NAc injection of SUL (KO + SUL, vs control + SUL; p > 0.9999, vs control + VEH; p > 0.9999), suggesting the involvement of dopamine D_2_-like receptor-mediated signaling in the antidepressant-like effects of the KO diet (p < 0.01, comparison of immobility for KO + VEH [*n* = 11] and the KO + SUL [*n* = 7] groups; Bonferroni comparisons). The reduction of immobility time was also abolished by microinjection of SKF into the NAc (KO + SKF, vs control + SKF; p > 0.9999, vs control + VEH; p > 0.9999, KO + VEH; p < 0.01). These results suggest that both types of dopamine receptors are involved in the antidepressant-like effects of the KO diet.

### KO diet increases tyrosine hydroxylase-positive cells in the VTA

**Fig 3:**
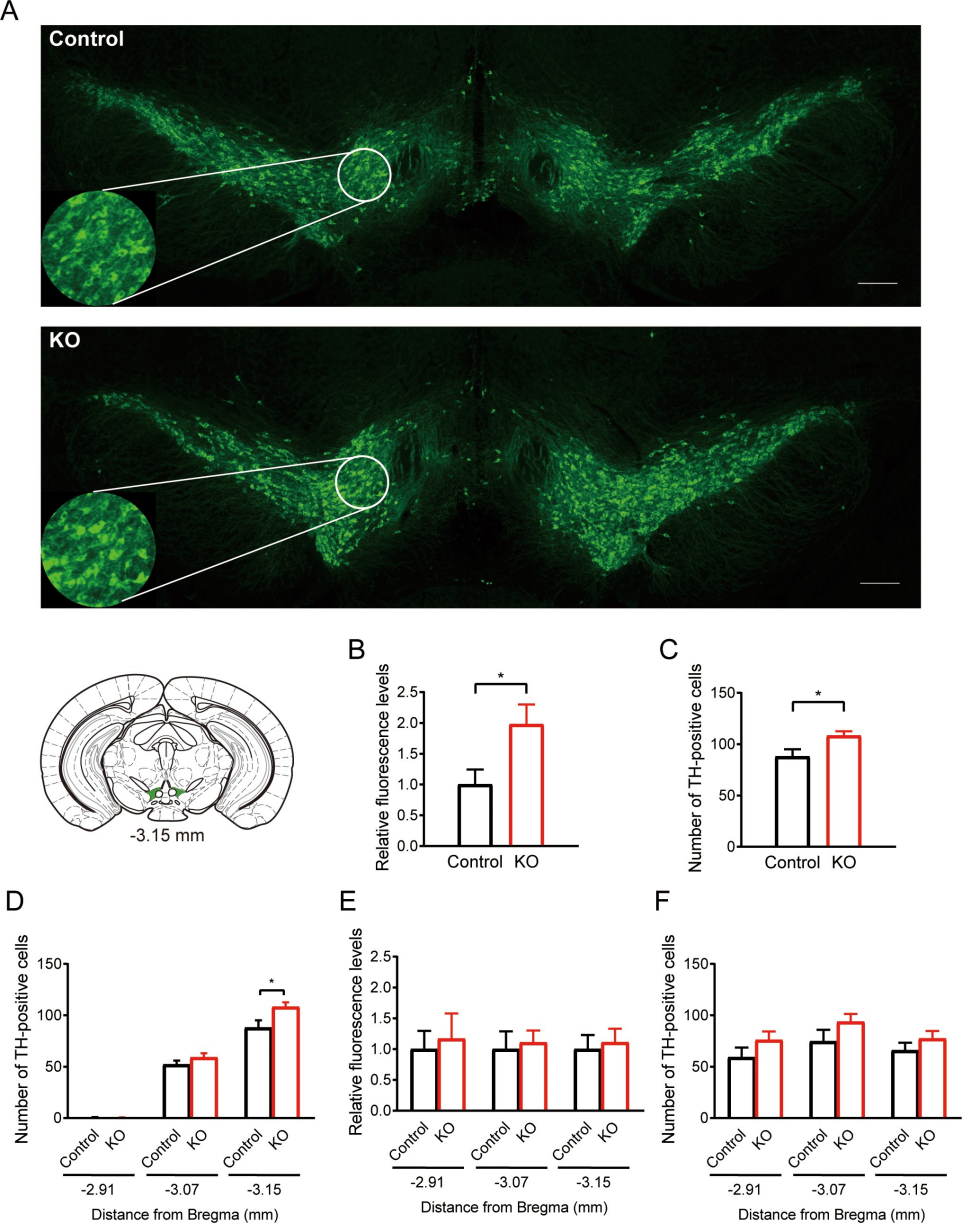
Effects of the KO diet on TH-positive cells in the midbrain. (A) Immunofluorescent staining of tyrosine hydroxylase (TH) in the ventral tegmental area (VTA) in the coronal sections at bregma -3.15 mm from mice fed the control diet (upper panel) and mice fed the KO diet (lower panel). The scale bars represent 200 μm. The circular panels indicate the area in which TH-positive cells were counted. (B, C) Comparison of the relative fluorescent levels (B) and the number of TH-positive cells (C) between the control and KO diet group at bregma -3.15 mm in the VTA. (D) Comparison of the number of TH-positive cells at different bregma levels in the VTA. The data at bregma -3.15 mm were copied from (C). (E, F) Comparison of relative fluorescent levels (E) and the number of TH-positive cells (F) at different bregma levels in the dorsal of the substantia nigra pars compacta (SNCD) between the control and the KO diet group. Data are obtained from two slices each from five mice. All data are presented as mean and SEM. *: p < 0.05.

Fig 3 shows TH-immunofluorescent staining in the VTA of male mice. Mice fed the KO diet exhibited higher TH-immunofluorescent levels than mice fed the control diet (two slices each from mice, n = 5 for each group, *U* [10, 10] = 17, p < 0.05; Fig 3B). We next counted TH-positive cells in the VTA, and we found that the number of TH-positive cells was greater in mice fed the KO diet than in mice fed the control diet (*t* [18] = 2.454, p < 0.05; Fig 3C). The increase in TH-positive cells was positioned in a bregma-dependent manner (*U* [10, 10] = 42, p = 0.628 for -2.91 mm, *U* [10, 10] = 34, p = 0.240 for -3.07 mm; Fig 3D). No significant differences in the relative TH-immunofluorescent levels (from bregma -2.91 mm: *U* [10, 10] = 49, p = 0.952; -3.07 mm: *t* [18] = 0.3022, p = 0.766; -3.15 mm: *t* [18] = 0.3398, p = 0.738; Fig 3E) and the number of TH-positive cells (from bregma -2.91 mm: *t* [18] = 1.339, p = 0.197; -3.07 mm: *t* [18] = 1.399, p = 0.179; -3.15 mm: *t* [18] = 1.098, p = 0.287; Fig 3F) in the dorsal tier of the substantia nigra pars compacta (SNCD) were found between these two groups. These results suggest that the KO diet enhanced TH expression in a bregma-dependent manner.

### Other behavioral tests

**Fig 4:**
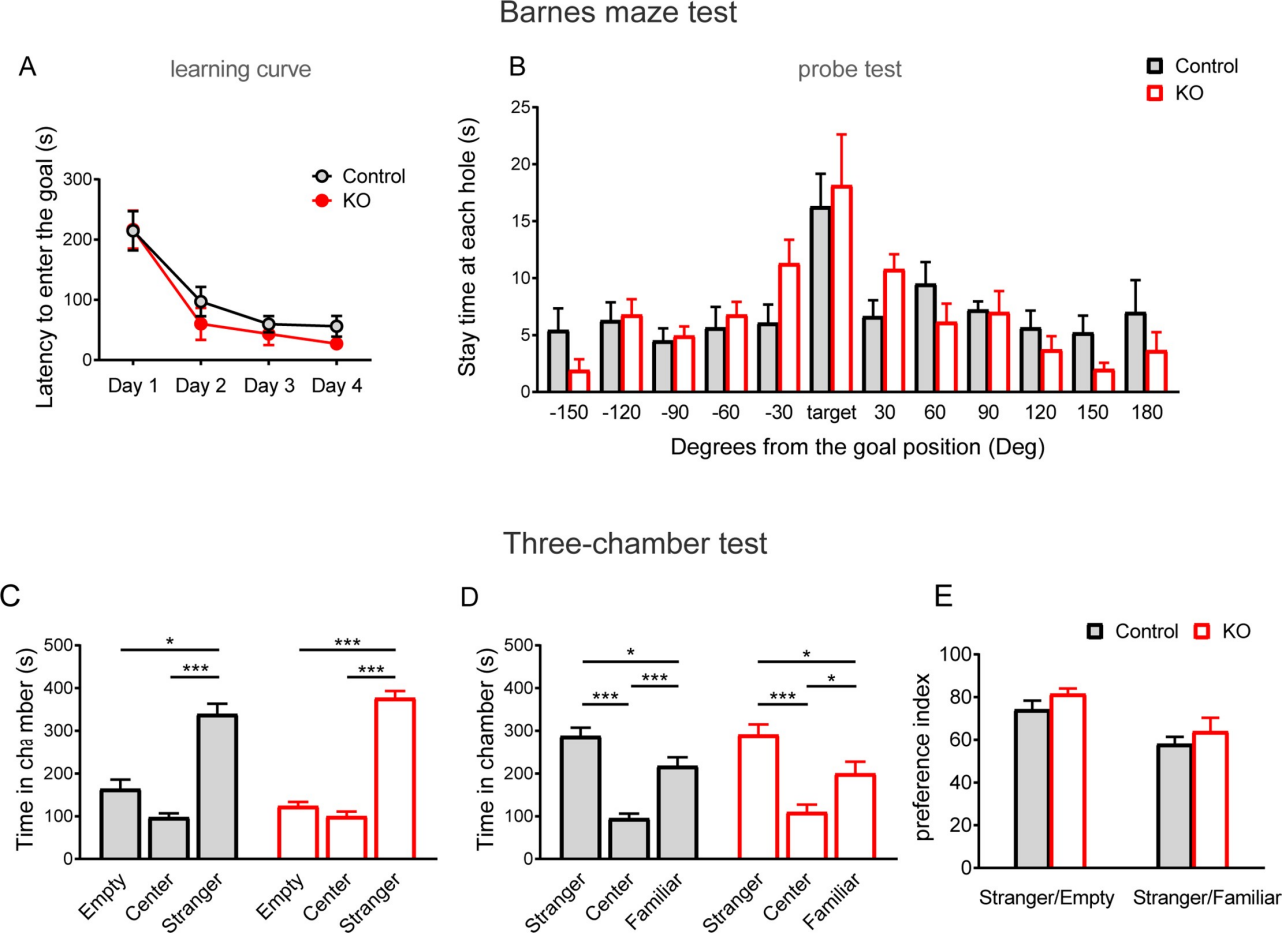
Spatial learning and memory and social behavior were not affected by the KO diet. (A, B) Spatial learning and memory performance in the Barnes maze test. (A) The learning curve during trainings (Day 1–4, 3 trials per day). No significant differences were detected in latency to get the goal box (*n* = 8 per group). (B) Stay time in each quadrant area during the test session (Day 5). Both groups showed similar performance. (C-E) Social behavior in the three-chamber test. (C) Stay time in each chamber (*n* = 8 per group). Mice preferred to stay in a stranger chamber in both the control and KO diet groups. (D) Stay times in each chamber (*n* = 8 per group). Mice preferred to stay in a stranger chamber in both the control and KO diet groups. (E) Preference index, comparison of the time spent around the stranger mouse cage vs. empty cage and the stranger mouse cage vs. familiar mouse cage, which indicated that both groups of mice preferred novel stranger mice. All data are presented as mean and SEM. *: p < 0.05, ***: p < 0.001.

We further examined the effects of KO diets on spatial learning and memory using a Barnes maze test. The latency to reach the correct hole above the escape box was not significantly different between the control and KO diet groups, and no significant changes in the learning time course were evident between the control and KO diet groups (diet: *F* [1, 12] = 0.64, p = 0.439; Days: *F* [3, 36] = 44.71, p < 0.001; diet × Days: *F* [3, 36] = 0.5004, p = 0.684; two-way repeated measures ANOVA; Fig 4A). In addition, there were no significant differences in post-probe trials (p > 0.05, *t*- test; Fig 4B) between the two diet groups. Furthermore, we investigated social interaction using a three-chamber test (Fig 4C–4E) and found that both groups showed similar behavior to the mice that preferred to stay in the stranger chamber in the sociability (control: p < 0.001, Kruskal-Wallis; KO: *F* [2, 21] = 2.567, p < 0.001, one-way ANOVA; Fig 4C) or social novelty preference tests (control: *F* [2, 21] = 1.006, p < 0.001; KO: *F* [2, 21] = 0.3035, p < 0.001, one-way ANOVA; Fig 4D). The preference index of sociability, as defined by the ratio of the time spent around the stranger mouse cage/empty cage, was higher in both the control and KO groups, and no significant differences were observed between the two groups (*U* [8, 8] = 20, p = 0.223, Fig 4E, left). The preference index of social novelty preference, as defined by the ratio of the time spent around the stranger mouse cage/familiar mouse cage, was higher in both the control and KO groups, and no significant differences were observed between two groups (*t* [14] = 0.807, p = 0.433, Fig 4E, right). In our previous study, we investigated the effects of KO diets in several other behavioral tests [29]; no significant differences were found in total moving distance in the open field, dark light latency, and light box stay time in the light-dark transition test, and alteration choice rate in the Y-maze test. However, we found that KO diets clearly reduced conditioned fear memory in contextual/toned fear conditioning [29, 36]. These results suggest that the effects of KO diets are limited to specific mouse behaviors.

## Discussion

In this study, we investigated the effects of two kinds of diets containing sufficient amounts of ALA with either a high- or low-supplementation of ω-3 PUFAs. The results showed that the KO diet reduced FST-immobility and TST-immobility when compared with the control diet. The reduction of FST-immobility was eliminated after changing the KO diet to the control diet. The DOPAC levels increased in the NAc in male mice fed a KO diet when compared to mice fed a control diet. The changes in the levels of dopamine and related metabolites were more pronounced in female mice than in male mice. An increase in dopamine and its metabolites was not observed in the mPFC, HPC, and AMY in both male and female mice fed KO diets. Therefore, we next focused on the NAc and carried out a pharmacological verification of the reduction in FST-immobility. The results showed that the reduction in the immobility disappeared with the intra-NAc-injection of an antagonist of D_2_-like receptors, SUL, or D_1_-like receptors, SKF. Furthermore, increased TH immunohistochemical staining in the VTA was observed in the mice fed a KO diet. Collectively, the present findings suggest that modulation of the VTA-NAc dopaminergic pathway is implicated in the mechanisms by which a diet high in ω-3 PUFAs reduces mouse immobility behavior in the FST.

Several previous studies have suggested that a high ω-3 PUFA diet consistently reduces the immobility behavior in the rodent FST [13–15], which is consistent with the present study. These findings (reduced immobility time) are evident in both male and female mice and may offer biological evidence for the treatment of female depressive symptoms with ω-3 PUFAs [8]. Accordingly, elucidating the mechanisms underlying the different effects resulting in reduced immobility between males and females might be important. Although sex hormones such as estrogen are suspected to have influenced the results of the present study, other systemic sex-dependent factors may also have contributed to the effects. For example, the body weight of female mice was consistently about 25% lower than that of male mice aged around 10 weeks. Such physical differences may affect the metabolism of the ingested ω-3 PUFAs.

To our knowledge, the present study is the first to show the involvement of the VTA-NAc dopaminergic pathway in the effects of a diet rich in ω-3 PUFAs on FST-immobility behavior. This result is consistent with that of a previous clinical study showing the robust relationship between ω-3 PUFA and the dopaminergic systems [23]. A previous study suggested that RXR is involved in DHA modulation of the immobility behavior in mice [17]. As RXR is a transcription factor that binds DHA, this receptor possibly contributes to the dietary role in FST-immobility behavior by changing the expression of genes that influence VTA dopaminergic neurons. Identification of such systems may facilitate the use of ω-3 PUFAs in mental health strategies. Another study suggests that the 5-HT1A receptor is involved in rat FST-immobility behavior [18]. Notably, in both male and female mice, we did not observe significant changes in serotonin, noradrenaline, and related metabolites in any of the four tested brain regions (the mPFC, NAc, HPC, and AMY). Nevertheless, the 5-HT1A receptor possibly contributes to the effect of the ω-3 PUFA diets in other brain regions.

In a previous study, rats fed ALA-deficient diets showed enhanced extracellular levels of dopamine and decreased levels of DOPAC and HVA in the NAc [19]. In the present study, the tissue levels of DOPAC were enhanced by ingestion of a high ω-3 diet in mice fed ALA-sufficient diets, suggesting that DOPAC plays an important role in the action of ω-3 PUFAs in male mice. However, it should be noted that we quantified the tissue levels of DOPAC in this study, but the extracellular levels of DOPAC were quantified in a previous study. In female mice, increased levels of dopamine, HVA, and 3-MT were observed in the NAc, suggesting that these metabolites may play an important role in the action of ω-3 PUFAs in female mice. Collectively, the NAc dopamine system is commonly altered in male and female mice after ingestion of diets rich in ω-3 PUFAs, but the underlying mechanism may be different between male and female mice. The different changes in dopamine or its metabolites may contribute to the different time course of behavioral effects between male and female mice.

The pharmacological results in the present study support the participation of both D_1_-like and D_2_-like dopamine receptors in the reduction in FST-immobility behavior. Recent findings suggest that both D_1_-like and D_2_-like receptors function through an NAc neuronal circuit; intra-NAc injection of D_1_-like antagonist or D_2_-like antagonist attenuates defensive treading behavior in rats [37]. In the present study, the complex neural circuits in the NAc were likely involved in this diet-induced action. Since dopaminergic neurotransmission from the VTA to the NAc is known to play an important role in emotional arousal and reward [38], we speculated that this pathway was activated after the ingestion of a diet high in ω-3 PUFAs to reduce FST-immobility. In the present study, the reason that TH-positive cells in the VTA are specifically affected by a diet high in ω-3 PUFAs was unclear. One plausible explanation was the presence of oxidative stress in dopaminergic cells. Hydrogen-peroxide is produced as a byproduct of dopamine metabolism and is then converted to the cell-toxic reactive oxygen species, hydroxyl radical [39]. DHA is known to enhance free radical scavenging and decrease lipid peroxidation in the rat fetal brain [40]. In addition, DHA attenuates oxidant-mediated endothelial cell injury [41]. It has been previously reported that KO inhibits lipopolysaccharide-induced oxidative stress [42]. Previous findings suggest the possibility that ω-3 PUFA acts as a radical scavenger to protect TH-positive cells from oxidative stress. Therefore, the increased TH-positive cell number likely reflects a lowered rate of cell death in the VTA of mice fed a ω-3 PUFA diet.

Data from the present and previous studies using KO diets suggested that significant changes in performance can be detected in the FST and TST, but not in the other behavioral tests, excluding fear conditioning [29]. In the present study, spatial learning and memory in the Barnes maze test was not significantly different between animals that ingested control and KO diets. In aged rats, administration of krill-phosphatidylserine significantly improves spatial learning in the Morris water maze [43]. Therefore, it should be noted that our experiments were carried out in adult mice younger than 13 weeks, but not in aged mice.

In summary, this study showed a reversible reduction in mouse immobility behavior with a KO diet under ALA-sufficient conditions. The present results indicate that the VTA-NAc dopaminergic pathways are changed in response to the ingestion of a KO diet. Both D_1_-like and D_2_-like receptors participate in the NAc neural circuits involved in the effect of this diet in the FST. Immunohistochemical analysis revealed that the number of TH-positive neurons was enhanced in the VTA of mice fed the KO diet. These results suggest that modulation of the VTA-NAc dopaminergic pathway is one mechanism through which this diet reduces mouse immobility behavior in the FST.

## Supporting information

S1 Table Levels of monoamine in the brain. (TIF)

S2 Table Monoamine turnover in the brain. (TIF)
