# Supplementary figures and images for: Participation of the nucleus accumbens dopaminergic system in the antidepressant-like actions of a diet rich in omega-3 polyunsaturated fatty acids
Source: PLoS One. 2020 Mar 25;15(3):e0230647. doi: 10.1371/journal.pone.0230647 (PMC7094879; doi:10.1371/journal.pone.0230647)

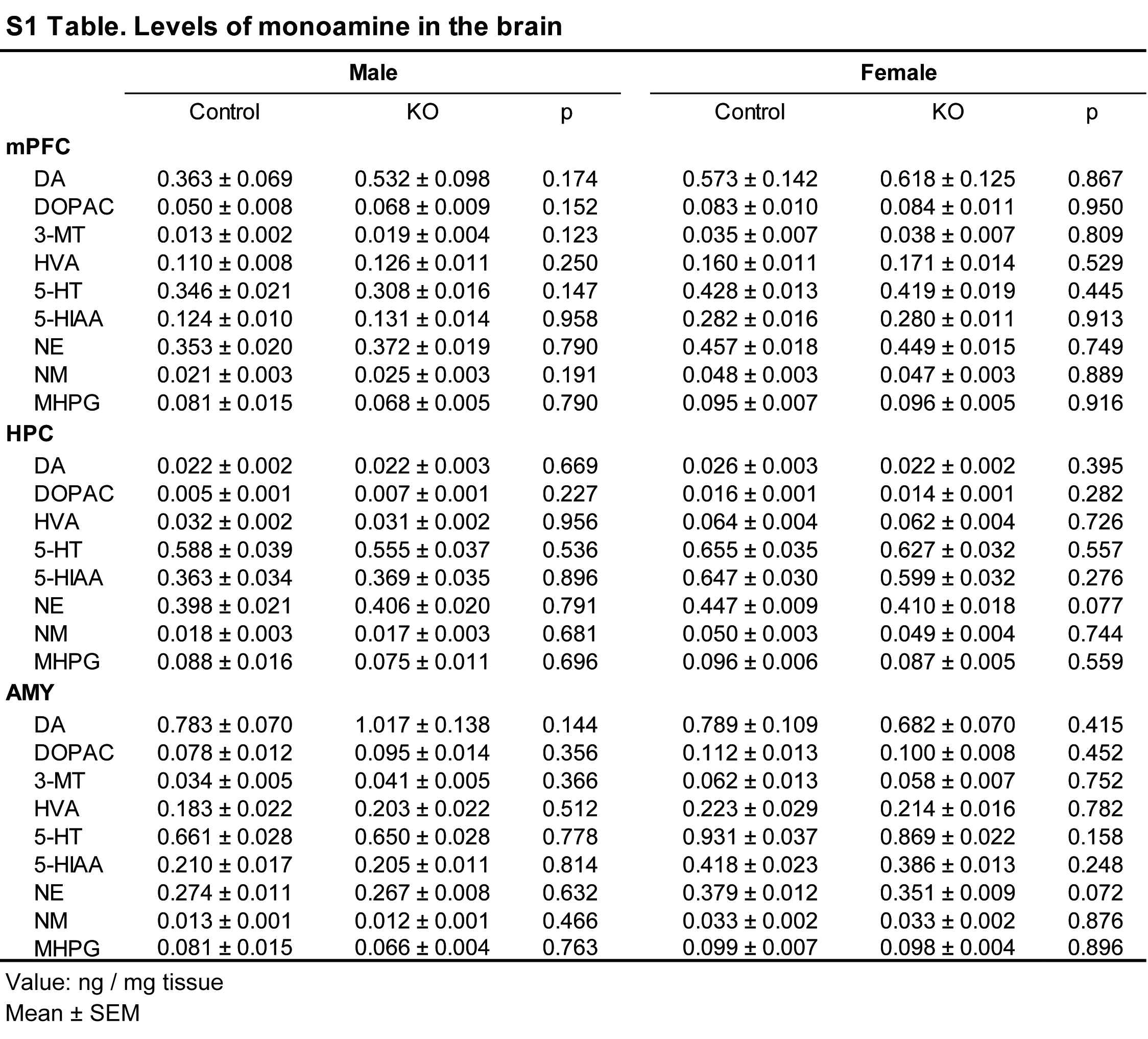

Supplement: S1 Table — (TIF) [file pone.0230647.s001.tif]

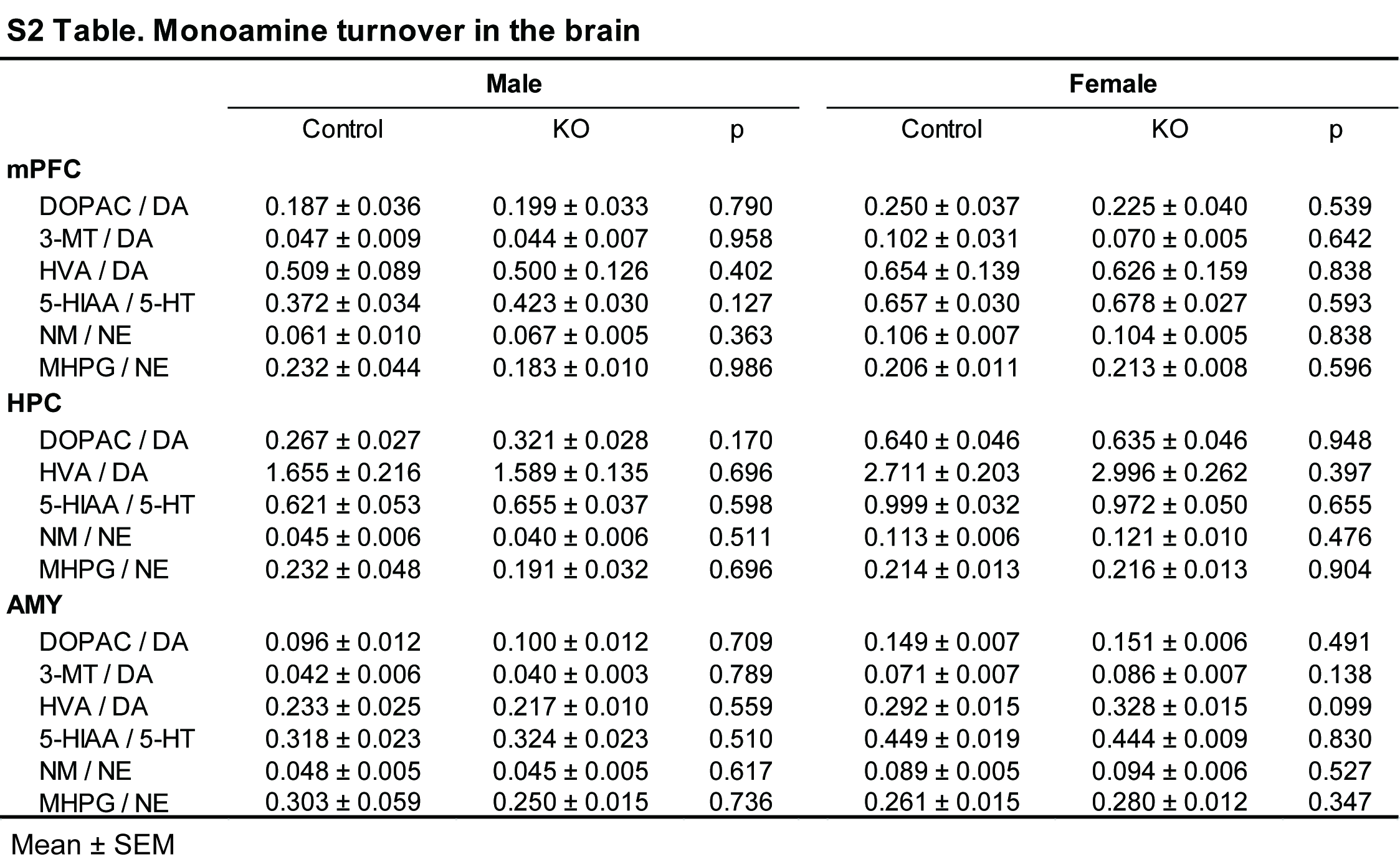

Supplement: S2 Table — (TIF) [file pone.0230647.s002.tif]
